# Sentinel Lymph Node Biopsy in Endometrial Cancer – A Systematic Review and Quality Assessment of Meta-Analyses

**DOI:** 10.1055/s-0042-1749067

**Published:** 2022-06-20

**Authors:** Mario Arturo González Mariño

**Affiliations:** 1 Department of Obstetrics and Gynecology, Faculty of Medicine, Universidad Nacional de Colombia, Bogotá, Colombia

**Keywords:** neoplasms, uterus, endometrium, sentinel lymph node, biopsy, endometrial neoplasms, neoplasias, útero, endométrio, linfonodo sentinela, biópsia, neoplasias endometriais

## Abstract

**Objective** To assess the quality of recent meta-analyses reviewing the diagnostic utility of sentinel node biopsy in endometrial cancer.

**Methods** With the MeSH terms *endometrial neoplasms* and *sentinel lymph node biopsy* , PubMed and Embase databases were searched on October 21, 2020, and again on November 10, 2021, with meta-analysis and publication date filters set to since 2015. The articles included were classified with the A Measurement Tool to Assess Systematic Reviews (AMSTAR 2) assessment tool.

**Results** The database searches found 17, 7 of which, after the screening, were selected for full review by the author, finally extracting six meta-analyzes for quality analysis. The rating with the AMSTAR 2 assessment tool found that overall confidence in their results was critically low.

**Conclusion** This study found that the quality of recent meta-analyses on the utility of the staging of endometrial cancer with sentinel node biopsy, evaluated by the AMSTAR 2 assessment tool, is classified as critically low, and, therefore, these meta-analyses are not reliable in the summary of their studies.

## Resumo

**Objetivo** Avaliar a qualidade de meta-análises recentes que revisaram a utilidade diagnóstica da biópsia do linfonodo sentinela no câncer de endométrio.

**Métodos** Com os termos MeSH *endometrial neoplasms* e *=biópsia do linfonodo sentinela* , as bases de dados PubMed e Embase foram pesquisadas em 21 de outubro de 2020 e novamente em 10 de novembro de 2021, com filtros de meta-análise e data de publicação configurados para desde 2015. Os artigos incluídos foram classificados com o instrumento de avaliação *A Measurement Tool to Assess Systematic Reviews* (AMSTAR 2).

**Resultados** As pesquisas de banco de dados encontraram 17 artigos, sete dos quais, após a triagem, foram selecionados para revisão completa pelo autor, extraindo finalmente 6 meta-análises para análise de qualidade. A classificação com a ferramenta de avaliação AMSTAR 2 descobriu que a confiança geral em seus resultados era criticamente baixa.

**Conclusão** Este estudo constatou que a qualidade de meta-análises recentes sobre a utilidade do estadiamento do câncer de endométrio com biópsia do linfonodo sentinela, avaliada pela ferramenta de avaliação AMSTAR 2, é classificada como criticamente baixa e, portanto, essas meta-análises não são confiáveis no resumo de seus estudos.

## Introduction

Endometrial cancer is the most common gynecological cancer in rich countries. 1 Overall survival is considered good because its diagnosis usually happens in the early stages, with the disease confined to the uterus, and surgery is often curative. 2

The standard surgical procedure, when indicated, is an extra-fascial total hysterectomy with bilateral salpingo-oophorectomy. 1 Lymphadenectomy is included for staging; it used to be performed in all cases, but, now, a more selective approach is preferred. 3 Node-positive documentation identifies a high-risk population and helps tailor adjuvant therapy for node-negative results, potentially reducing the need for external radiation therapy. 4 The therapeutic utility of lymphadenectomy is controversial; two randomized controlled trials showed no therapeutic benefit in early endometrial cancer, 5 6 but, instead, it is associated with significant morbidity, up to a 50% risk of lymphedema, 7 increased risk of bleeding, intraoperative injury, and increased surgical time. 8 Sentinel node biopsy offers relevant information, and it is a useful procedure to determine lymph node involvement in cases of early endometrial cancer, 3 9 with a lower risk of lymphedema. 9

To evaluate the quality of each meta-analysis included in this study, the A Measurement Tool to Assess Systematic Reviews (AMSTAR 2) tool, which allows critical evaluation of systematic reviews that include randomized or non-randomized studies as well as those with both designs in care interventions, was used. 10 This instrument considers that all its items used to assess systematic reviews are important, but that seven of them can critically affect the validity of a review and its conclusions. These items correspond to the existence of a protocol registered before the beginning of the review, adequate bibliographic search, justification for the exclusion of each of the studies, the risk of bias of each study included in the review, suitability of the methods of meta-analysis, consideration of risk of bias when interpreting the results of the review, and assessment of the presence and possible impact of publication bias. 10

## Methods

A search of publications was conducted using the MeSH terms *endometrial neoplasm* and *sentinel lymph node biopsy* in the PubMed and Embase databases on October 21, 2020, and, again, on November 10, 2021, with the filters of meta-analysis and publication date set to since 2015. The retrieved articles were screened by the title and abstract independently, with another evaluator agreeing to read the entire article in case of discrepancy and make their decision after this reading. The articles selected for this screening were studied by the author, who read the complete articles and determined their relevance for the review; those that were finally extracted were classified with the AMSTAR 2 evaluation tool.

## Results

**Fig. 1:**
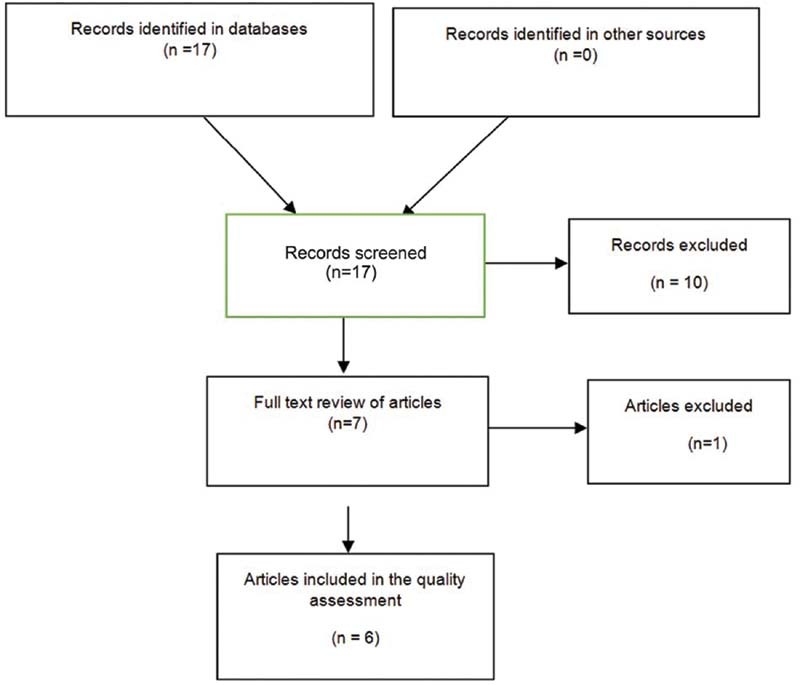
Information flow through the different phases of the systematic review.

The database searches found 17 articles, 7 of which were selected, after the screening, for full review by the author. Finally, six of them were included for quality analysis. Figure 1 shows that the excluded publication did not report the results of the sentinel node biopsy in endometrial cancer separately (the results were combined with those for cervical cancer). 11

A meta-analysis that included prospective cohort studies to evaluate sentinel lymph node biopsy in stage I high-grade endometrial cancer patients found a false negative rate of 8% (95% confidence interval [CI], 4–16%). 12 Other results of this study, as well as those of a study in laparoscopic surgery, 13 and two meta-analyses from 2017 14 15 are shown in Chart 1 . The study by Lin et al. 14 also evaluated the laparoscopic surgery subgroup that had the best sensitivity within the sentinel node mapping surgical options with 96% (95% CI: 88–99%).

**Chart 1:** Meta-analyses reporting the detection rate and sensitivity of sentinel node biopsy in endometrial cancer

Author	Detection rate (%) (95% CI)	Sensitivity (%) (95% CI)
Marchocki et al. 12	91 (85–95)	92 (84–96)
Wang and Liu 13	96 (95–98)	96.3 (94–98)
Lin et al. 14	83 (80–86)	91 (87–95)
Bodurtha Smith et al. 15	81 (77–84)	96 (91–98)

Abbreviation: CI, confidence interval.

Sentinel node biopsy was superior to lymphadenectomy in detecting positive pelvic nodes, but there was no difference in detecting positive para-aortic nodes in two meta-analyzes that analyze this issue. 16 17 Chart 2 . The classification of the items with the AMSTAR 2 assessment tool are shown for each study in Chart 3 .

**Chart 2:** Detection of pelvic and para-aortic nodes comparing sentinel node biopsy with lymphadenectomy in endometrial cancer

Author	Pelvic nodes Odds ratio (95% CI)	Paraortic nodes Odds ratio (95% CI)
Gu et al. 16	2.00 (1.21–3.32); *p* = 0.007	0.62 (0.24–1.64); *p* = 0.34
Bogani et al. 17	2.03 (1.30–3.18); *p* = 0.002	0,93 (0,39–2.18); *p* = 0.86

Abbreviation: CI, confidence interval.

**Chart 3:** Assessment of each domain in the meta-analyses rated with the critical evaluation tool for reviews AMSTAR 2

Question/author	1	2	3	4	5	6	7	8	9	10	11	12	13	14	15	16	Classification
Marchocki et al. 12	Y	Y	N	N	Y	N	Y	Y	N	N	N	N	N	N	N	Y	CL
Wang and Liu 13	Y	N	N	N	Y	Y	N	N	P	N	Y	Y	N	N	Y	Y	CL
Lin et al. 14	N	N	N	N	Y	Y	N	P	P	N	N	N	N	N	N	N	CL
Bodurtha Smith et al. 15	N	Y	N	N	Y	Y	N	Y	P	N	N	N	Y	Y	N	Y	CL
Gu et al. 16	Y	Y	N	N	Y	Y	Y	N	N	N	N	N	N	N	N	Y	CL
Bogani et al. 17	Y	P	N	P	N	N	Y	Y	N	N	N	N	N	N	N	Y	CL

Abbreviations: CL, critically low; N, no.; P, partial yes; Y, yes

## Discussion

Most patients with endometrial cancer present without lymph node metastases, with tumor confined to the uterus (about 75% stage I of the International Federation of Gynecology and Obstetrics [FIGO] classification) that has a rate of overall survival greater than 90%. 18

The acceptance of sentinel node mapping within the National Comprehensive Cancer Network (NCCN) guidelines as a procedure to be considered in the surgical staging of endometrial cancer apparently confined to the uterus, without evidence of metastasis in the images and without evidence of extrauterine disease in surgery, 3 confirms the indication of this procedure in surgical practice given the difficulty in selecting cases for lymphadenectomy, as well as the lack of benefit in early stages when this surgical procedure is performed, evidenced in randomized studies, and its high rate of complications. Sentinel node mapping can allow staging with a simple, rapid procedure and a lower risk of complications. 1 However, the speed of its acceptance does not seem consistent with the currently available evidence. The inclusion of sentinel node mapping in endometrial cancer in clinical practice has a low level of evidence derived mainly from observational studies, and it is desirable to have more randomized studies to support its acceptance as an alternative in the staging of this pathology. However, it is a story that begins to seem to the current standard use of the sentinel node in the staging of axillary nodes in clinically node-negative early breast cancer, ^19^ in which its use was extended to the clinical setting, without high-level studies, despite the insistence on the need for randomized studies but that was able to demonstrate their advantages in the following years. 20 For greater safety with this new surgical option, it is recommended that surgeons developing this technique adhere to an algorithm that includes a thorough evaluation of retroperitoneal lymph nodes, selective or side-specific lymphadenectomy, if there is no identified mapping within a hemipelvis, and removal of all suspicious lymph nodes regardless of the mapping. 21

The quality evaluation of each study found the general confidence of their results to be critically low according to the AMSTAR 2 tool. This means that the review has more than one critical flaw and should not be relied upon to provide an accurate and complete summary of the available studies. 10 Among the critical domains, those corresponding to items 9 and 13 of the AMSTAR 2 listing refer to the risk of biases, which are present in all the meta-analyses evaluated here in different magnitudes, except for the one by Bodurtha Smith et al. 15 for the consideration of these risks in the analysis of the results of the review. These items that assess the risk of bias are given priority in the classification because of the inclusion in the reviews of non-randomized studies.

Meta-analyses are important components of scientific information in evidence-based medicine. 22 The number of these reviews has increased steadily, but their quality has not always kept pace with this number. 23 To this issue, many instruments have been designed to evaluate the different aspects of a review, AMSTAR 2 allows a more detailed evaluation of systematic reviews that include non-randomized studies, which are increasingly being incorporated into these studies. 10

The limitations of this study are due to the design of the AMSTAR 2 tool in the evaluation of the planning and performance of the reviews. As a new tool that includes non-randomized studies in systematic reviews, it is necessary to wait for the feedback of users of the instrument to consider making modifications. 10

## Conclusion

The current study found that the quality of recent meta-analyses on the utility of sentinel node biopsy in the staging of endometrial cancer, evaluated by the AMSTAR 2 assessment tool — which allows evaluating systematic reviews that include non-randomized studies — is classified as critically low, and, therefore, these meta-analyses are not reliable to be used in the summary of their studies.
